# Bioclimatic and anthropogenic variables shape the occurrence of *Batrachochytrium dendrobatidis* over a large latitudinal gradient

**DOI:** 10.1038/s41598-021-96535-w

**Published:** 2021-08-30

**Authors:** Mario Alvarado-Rybak, Manuel Lepe-Lopez, Alexandra Peñafiel-Ricaurte, Andrés Valenzuela-Sánchez, Catalina Valdivia, Fernando O. Mardones, Leonardo D. Bacigalupe, Robert Puschendorf, Andrew A. Cunningham, Claudio Azat

**Affiliations:** 1 Sustainability Research Centre & PhD Programme in Conservation Medicine, Life Sciences Faculty, Universidad Andres Bello, Republica 252, Santiago, Chile; 2 Institute of Zoology, Zoological Society of London, Regent’s Park, London, NW1 4RY UK; 3 Núcleo de Ciencias Aplicadas en Ciencias Veterinarias y Agronómicas, Universidad de las Américas, Echaurren 140, Santiago, Chile; 4 ONG Ranita de Darwin, Nataniel Cox 152, Santiago, Chile; 5 Instituto de Conservación, Biodiversidad y Territorio, Facultad de Ciencias Forestales y Recursos Naturales, Universidad Austral de Chile, 5110566 Valdivia, Chile; 6 Escuela de Medicina Veterinaria, Facultad de Agronomía e Ingeniería Forestal, Facultad de Ciencias Biológicas y Facultad de Medicina, Pontificia Universidad Católica de Chile, Santiago, Chile; 7 Instituto de Ciencias Ambientales y Evolutivas, Facultad de Ciencias, Universidad Austral de Chile, Valdivia, Chile; 8 School of Biological and Marine Sciences, University of Plymouth, Plymouth, PL4 8AA UK

**Keywords:** Biodiversity, Conservation biology, Fungal infection

## Abstract

Amphibian chytridiomycosis, caused by the fungus *Batrachochytrium dendrobatidis* (*Bd*), has caused the greatest known loss of biodiversity due to an infectious disease. We used *Bd* infection data from quantitative real-time PCR (qPCR) assays of amphibian skin swabs collected across Chile during 2008–2018 to model *Bd* occurrence with the aim to determine bioclimatic and anthropogenic variables associated with *Bd* infection. Also, we used *Bd* presence/absence records to identify geographical *Bd* high-risk areas and compare *Bd* prevalence and infection loads between amphibian families, ecoregions, and host ecology. Data comprised 4155 *Bd*-specific qPCR assays from 162 locations across a latitudinal gradient of 3700 km (18º to 51ºS). Results showed a significant clustering of *Bd* associated with urban centres and anthropogenically highly disturbed ecosystems in central-south Chile. Both *Bd* prevalence and *Bd* infection loads were higher in aquatic than terrestrial amphibian species. Our model indicated positive associations of *Bd* prevalence with altitude, temperature, precipitation and human-modified landscapes. Also, we found that macroscale drivers, such as land use change and climate, shape the occurrence of *Bd* at the landscape level. Our study provides with new evidence that can improve the effectiveness of strategies to mitigate biodiversity loss due to amphibian chytridiomycosis.

## Introduction

In recent years, amphibians have declined dramatically in many regions of the world^1^ with approximely 50% of amphibian species under risk of global extinction^2,3^. The causes behind these declines are multiple and complex, and they include well-established factors such as habitat loss and invasive species and, more recently, infectious diseases^4,5^. The discovery of the emerging disease amphibian chytridiomycosis^6^, caused by the chytrid fungus *Batrachochytrium dendrobatidis* (hereafter *Bd*)^7^, and its role in the decline and extinction of numerous amphibian species has led to a paradigm shift towards wildlife diseases as a conservation issue^8^. Recently, another species, *B. salamandrivorans*, has been found causing severe mortality and population declines of fire salamanders (*Salamandra salamandra*) in Europe^9^. Thus far, *Bd* has been associated with the decline of over 500 amphibian species, including the presumed extinction of 90 species^10^ (e.g., but see^11,12^), and is linked with the collapse of amphibian communities in eastern Australia^6^, Costa Rica^13,14^, Panama^4,15,16^ and Peru^17^. The fungus infects amphibian skin, leading to epidermal hyperplasia and hyperkeratosis, resulting in death in susceptible individuals due to electrolyte loss and osmotic imbalance^18^. The impacts of *Bd* on amphibian populations can be attributed to the introduction of this pathogen into naïve host populations, the persistence of *Bd* in the environment, the existence of a free-living infective stage, and the presence of amphibian reservoir hosts^8,19,20^.

Studies analysing the distribution patterns of *Bd* at the large scale show a broad spatial distribution, high environmental tolerance, and a wide range of host species, indicating that *Bd* is a generalist pathogen^21–27^. Several factors related to host^28,29^, pathogen^30^, and environment^21^ have been shown to interact, facilitating the emergence of *Bd* and increasing the severity of its impacts. However, many aspects of the landscape epidemiology of *Bd* (i.e., studies comprising different ecoregions) remain unknown. These include pathogen distribution in under-surveyed areas (e.g., parts of Africa and South America), mechanisms for local or regional spread and identification of reservoir hosts (amphibian and non-amphibian)^8,14,31–33^. These can be relevant for *Bd* mitigation, for example helping to predict the potential impacts of chytridiomycosis in understudied areas or following *Bd* introduction into naïve amphibian populations. Also, *Bd* landscape epidemiology is crucial to inform biosecurity recommendations at the country level, particularly given the potential for strain recombination to result in increased virulence^8^. Among climatic factors, temperature and humidity have been reported to be important determinants of *Bd* occurrence, influencing the survival and infection rate of the pathogen^19,24,25,34–36^. Chytridiomycosis outbreaks generally have been associated with cooler months and higher altitudes^4,23,37,38^. Seasonal climatic variation can affect the occurrence of the pathogen and the timing of chytridiomycosis outbreaks in the wild^13,35^; for example, infection prevalence in robber frogs (*Eleutherodactylus* spp.) in Montserrat is higher in the cool, dry season than in the warmer, wetter months^39^. This is also of interest in temperate areas where climate has a strong seasonality, which is also influenced by latitude and altitude^40–42^. In addition, urbanization has been proposed as a factor associated with high *Bd* occurrence^24,36^. Nevertheless, we believe that understanding of the factors driving the occurrence of *Bd* infection have been overlooked, despite the fundamental answers that such insight may provide, particularly focused on disease mitigation strategies^43^. Taking a regional perspective (in contrast to a global or local one) might allow the use of high-resolution data over a large spatial scale (such as the case of Chile) to evaluate potential interactions among different factors involved in the epidemiology of *Bd*.

South America is one the regions most impacted by amphibian chytridiomycosis^7,44,45^, and *Bd* has been detected in wild amphibians in virtually all countries in this region^25^. Here, amphibian population declines, and extinctions have been reported since the 1970s^5^. A likely recent introduction of the hypervirulent Global Panzootic Lineage of *Bd* (*Bd*GPL) into South America^46,47^ coincides with the onset of these enigmatic amphibian declines^5,19,48^, but the presence in a restricted area of the Atlantic forests of Brazil of the more ancient lineage *Bd*Asia-2/*Bd*Brazil could make the evolutionary history of *Bd* in in this region more complex^47^. Native Chilean amphibians consist of 63 anuran species, of which 45 (72%) are endemic, and adapted to a range of different ecosystems, from dry desert and altiplano in the North, to subpolar forest and cold steppe in the south^49^. In addition, feral populations of the African clawed frog (*Xenopus laevis*) have been established in central Chile since the 1970s^50^. *Batrachochytrium dendrobatidis* infection is widespread in Chile^20,36,43,51,52^, and chytridiomycosis has been associated with the population decline and extinction of Darwin’s frogs (*Rhinoderma rufum* and *R. darwinii*)^52,53^.

Here, we use *Bd* occurrence data of amphibians from across Chile collected over 11 years to assess the epidemiology of *Bd* over time and across a large latitudinal, altitudinal and taxonomic range. Based on the results of quantitative real-time PCR (qPCR) assays, we modelled *Bd* prevalence to determine bioclimatic and anthropogenic variables associated with the distribution of infection. Using *Bd* presence/absence records we followed a spatial scan statistics approach to identify geographical *Bd*-infection clusters (or *Bd* high-risk areas). Finally, we compare variation in *Bd* prevalence and infection loads among taxonomic families, ecoregions and host ecology (aquatic vs terrestrial, see^14,54^) to further complement our epidemiological study. Although similar studies have been conducted previously^40,43,55,56^, our multi-approach analyses using different methods over such a large latitudinal gradient has not been done before, and thus can provide new insights into the landscape epidemiology of *Bd*. Understanding macroscale drivers of *Bd* and their interactions is critical to the conservation management of amphibians through *Bd* prevention and mitigation strategies^31^.

## Results

### *Bd* prevalence patterns

**Figure 1:**
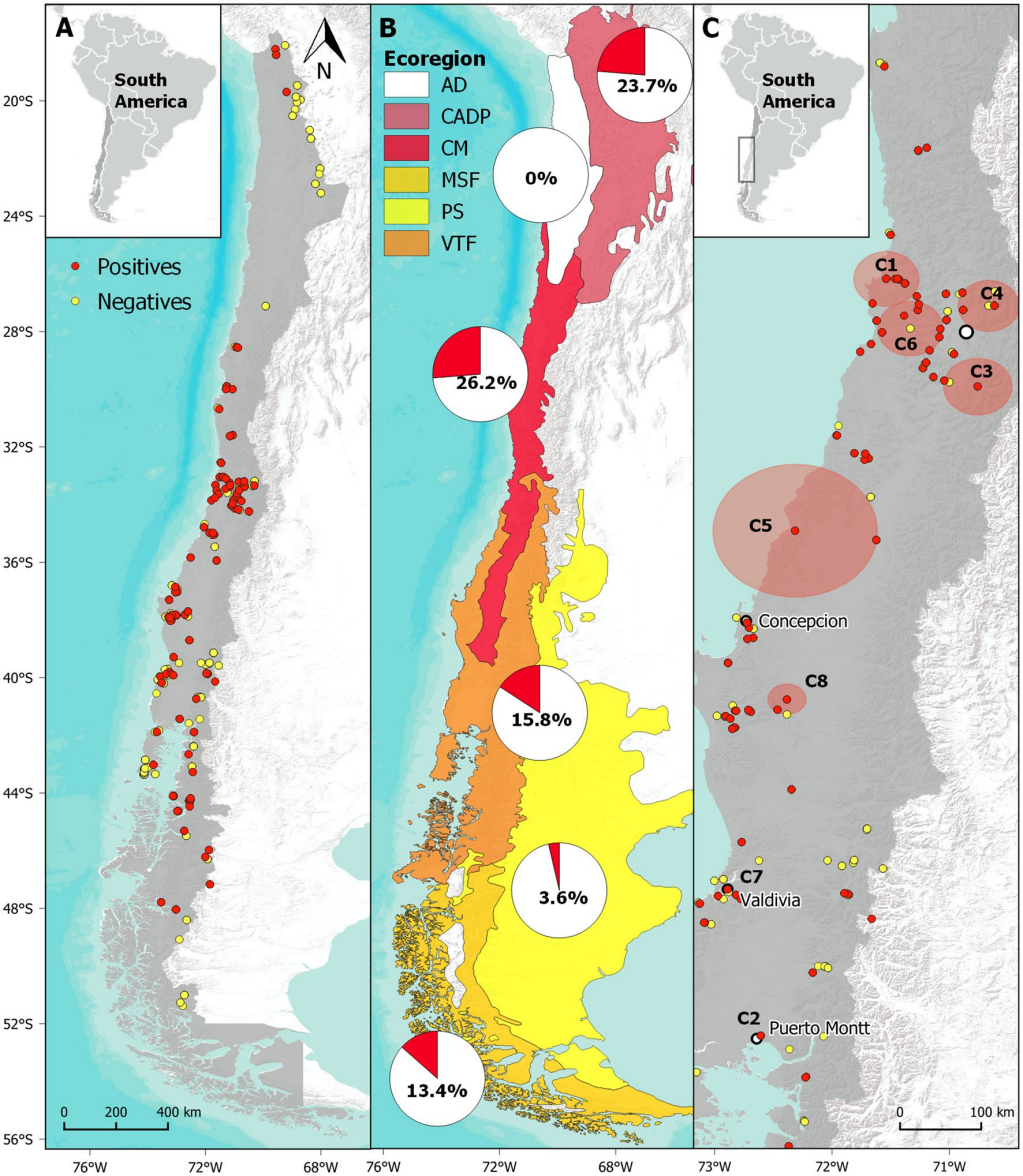
Spatial distribution of *Batrachochytrium dendrobatidis* (*Bd*) infection in amphibian populations in Chile studied from 2008 to 2018. (**A**) *Bd*-positive (red dots) and *Bd*-negative (yellow dots) sites. (**B**) Proportion of *Bd*-positive amphibians (red portion of pie chart) according to ecoregions: Chilean Andean Dry Puna (CADP), Atacama Desert (AD), Chilean Matorral (CM), Valdivian Temperate Forest (VTF), Patagonian Steppe (PS) and Magellanic Subpolar Forest (MSF). (**C**) Eight statistically significant (*P* < 0.05) spatial clusters of *Bd* high-risk areas (C1 to C8) obtained from scan statistics analysis. All clusters were located in central and south Chile. Most clusters are represented by red shaded circles, apart from C2 and C7 which were smaller in size. Maps and rasters were generated using QuantumGIS v.3.8.2 (QGIS Geographic Information System). Open Source Geospatial Foundation Project. http://qgis.osgeo.org (2018).

In total, we processed swab samples from 4155 wild anurans collected over 11 years (2008–2018) from 40 species across Chile (Fig. 1, Table 1). Infection with *Bd* was detected across a broad geographical range (97 out of 162 sites were infected) with an overall prevalence of 19.1% (95% CI 17.8–20.3). The prevalence of *Bd* infection varied throughout the 11 years of the study (GLM, d.f. = 47, *P* < 0.05), with the odds ratio analysis showing that anurans are more likely to get infected by *Bd* over time (OR = 1.03, 95% CI 1.01–1.07). The Chilean Matorral ecoregion had the highest prevalence of *Bd* infection (26.2% [95% CI 23.9–28.7%], GLM, d.f. = 47, OR = 6.2, 95% CI 4.9–7.7, *P* < 0.05) in comparison with the other studied ecoregions (Fig. 1).

**Table 1:** Chilean anurans studied for *Batrachochytrium dendrobatidis* (*Bd*) infection from 2008 to 2018.

Species	Ecology	Negative	Positive	Sample size	Proportion of infection (%)	95% CI	Median ZE	IUCN
*Alsodes australis*	T	13	0	13	0	0–22.8	0	DD
*Alsodes barrioi*	T	27	0	27	0	0–12.5	0	EN
*Alsodes coppingeri*	T	2	0	2	0	0–65.8	0	DD
*Alsodes nodosus*	A	27	5	32	15.6	6.9–31.8	166	NT
*Alsodes tumultuosus*	A	47	21	68	30.9	21.2–42.6	1302	VU
*Alsodes valdiviensis*	T	11	9	20	45.0	25.8–65.8	13	EN
*Alsodes verrucosus*	T	3	0	3	0	0–56.2	0	EN
*Atelognathus salai*	A	0	4	4	100	51.0–100	419	LC
*Batrachyla antartandica*	A	143	21	164	12.8	8.5–18.8	62	LC
*Batrachyla leptopus*	A	66	2	68	2.9	0.8–10.1	1.6	LC
*Batrachyla taeniata*	A	83	5	88	5.7	2.5–12.6	318	LC
*Calyptocephalella gayi*	A	281	150	431	34.8	30.5–39.4	436	VU
*Chaltenobatrachus grandisonae*	A	22	8	30	26.7	14.2–44.5	621	LC
*Eupsophus altor*	T	5	0	5	0	0–43.5	0	NE
*Eupsophus calcaratus*	T	87	12	99	12.1	7.1–20.0	53	LC
*Eupsophus contulmoensis*	T	30	7	37	18.9	9.5–34.2	30	EN
*Eupsophus emiliopugini*	A	17	1	18	5.6	0.3–25.8	6	LC
*Eupsophus migueli*	T	1	0	1	0	0–94.9	0	EN
*Eupsophus nahuelbutensis*	T	104	5	109	4.6	2.0–10.3	55	EN
*Eupsophus roseus*	A	35	4	39	10.3	4.1–23.6	86	LC
*Eupsophus septentrionalis*	A	10	2	12	16.7	4.7–44.8	33	DD
*Eupsophus vertebralis*	T	9	0	9	0	0–29.9	0	LC
*Hylorina sylvatica*	T	6	3	9	33.3	12.1–64.6	222	LC
*Nannophryne variegata*	T	3	0	3	0	0–56.2	0	LC
*Pleurodema bufonina*	A	98	6	104	5.8	2.7–12	154	LC
*Pleurodema thaul*	A	737	266	1003	26.5	23.9–29.3	115	LC
*Rhinella arunco*	T	4	11	15	73.3	48.1–89.1	61	NT
*Rhinella atacamensis*	T	26	0	26	0	0–12.9	0	VU
*Rhinella spinulosa*	T	45	0	45	0	0–7.9	0	LC
*Rhinoderma darwinii*	T	788	14	802	1.8	1.0–2.9	1170	EN
*Telmatobius chusmisensis*	A	11	24	35	68.6	52.0–81.5	1830	EN
*Telmatobius dankoi*	A	49	0	49	0	0–7.3	0	CR
*Telmatobius fronteriensis*	A	14	0	14	0	0–21.5	0	CR
*Telmatobius marmoratus*	A	31	0	31	0	0–11.0	0	EN
*Telmatobius pefauri*	A	19	16	35	45.7	30.5–61.8	28	CR
*Telmatobius peruvianus*	A	5	6	11	54.6	28.0–78.7	2	VU
*Telmatobius halli*	A	7	0	7	0	0–35.4	0	DD
*Telmatobius vilamensis*	A	13	0	13	0	0–22.8	0	CR
*Telmatobufo bullocki*	A	3	0	3	0	0–56.2	0	EN
*Xenopus laevis*	A	388	172	560	30.7	27.0–34.7	400	LC

Host ecology (aquatic = A, or terrestrial = T), positive, negative, sample size, proportion of Bd infection, and 95% binomial confidence intervals (CI). Bd infection loads are shown as median zoospore equivalents per swab (ZE). Conservation status for each species from the IUCN redlist is also showed.

**Figure 2:**
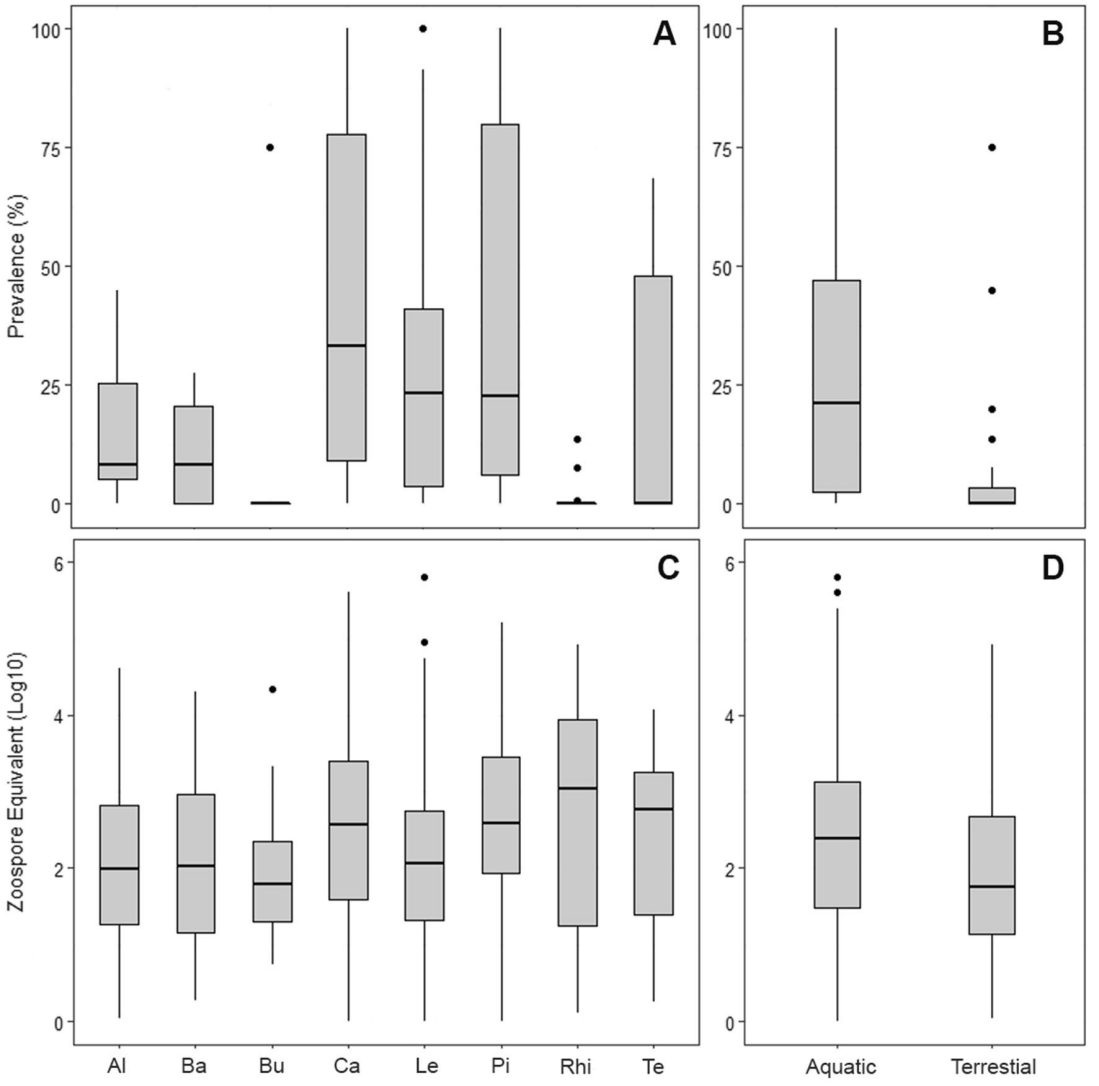
Boxplots showing *Batrachochytrium dendrobatidis* (Bd) prevalence (%) by (**A**) anuran family and (**B**) host ecology (aquatic vs. terrestrial), and Bd zoospore equivalent (Log_10_) by (**C**) anuran family and (**D**) host ecology, for amphibians in Chile collected from 2008 to 2018. Anuran family abbreviation: Al (Alsodidae), Ba (Batrachylidae), Bu (Bufonidae), Ca (Calyptocephalellidae), Le (Leptodactylidae), Pi (Pipidae), Rhi (Rhinodermatidae) and Te (Telmatobiidae). The centre line in each box indicates the median, the upper and lower box sides represent the interquartile range, the whiskers extend to the 5th and 95th percentiles and dots represent outliers.

Among the eight anuran families present in Chile, Calyptocephalellidae showed the highest *Bd* infection prevalence with 34.5% (95% CI 30.2–39.1, GLM, d.f. = 47, OR = 1.13, 95% CI 0.7–1.8, *P* = 0.6), followed by Pipidae with 30.7% (CI 27.4–34.6, GLM, d.f. = 47, OR = 1.6, 95% CI 1–1.8, *P* < 0.05) and Leptodactylidae with 24.5% (95% CI 22.1–27.1; Fig. 2A). Sixty percent (24 out of 40) of the sampled anuran species had at least one individual positive for *Bd* infection (Table 1). The species with the highest prevalence were *Telmatobius chusmisensis* with 68.6% (95% CI 52–81.5), *T. pefauri* with 45.7% (95% CI 30.5–61.8) and *C. gayi* with 34.8% (95% CI 30.4–39.4). Aquatic anurans exhibited a significantly higher prevalence of 25.3% (95% CI 23.7–26.9) than terrestrial anurans with 4.9% (95% CI 3.9–6.34; GLM, d.f. = 47, OR = 4.3, 95% CI 2.5–7.5, *P* < 0.05; Fig. 2B).

For each species sampled, the proportion of swabbed animals that were *Bd*-positive and the median infection load are summarized in Table 1. The infection load in *Bd*-positive amphibians ranged from 0.1 to 630,720 ZE (Zoospore equivalents; median = 234; the highest load was obtained from a *P. thaul* individual). Despite the presence of some high infection loads, swabbed animals did not exhibit clinical signs consistent with chytridiomycosis. Of the total number of infected frogs, only 6.5% (774 individuals) had more than 10,000 ZE/swab. There were no significant differences in *Bd* infection load across ecoregions (GLM, d.f. = 47, *P* = 0.7), but at the family level, Rhinodermatidae, Calyptocephalellidae, and Telmatobiidae had higher burdens of infection compared with other families (GLM, d.f. = 47, *P* < 0.01; Fig. 2C). Overall, aquatic anurans had greater *Bd* infection loads (median = 259.2; range: 0.1–630,720) compared with terrestrial species (median = 56.4; range: 0.1–84,709; GLM, d.f. = 47, *P* < 0.01; Fig. 2D).

### *Bd* modelling

**Figure 3:**
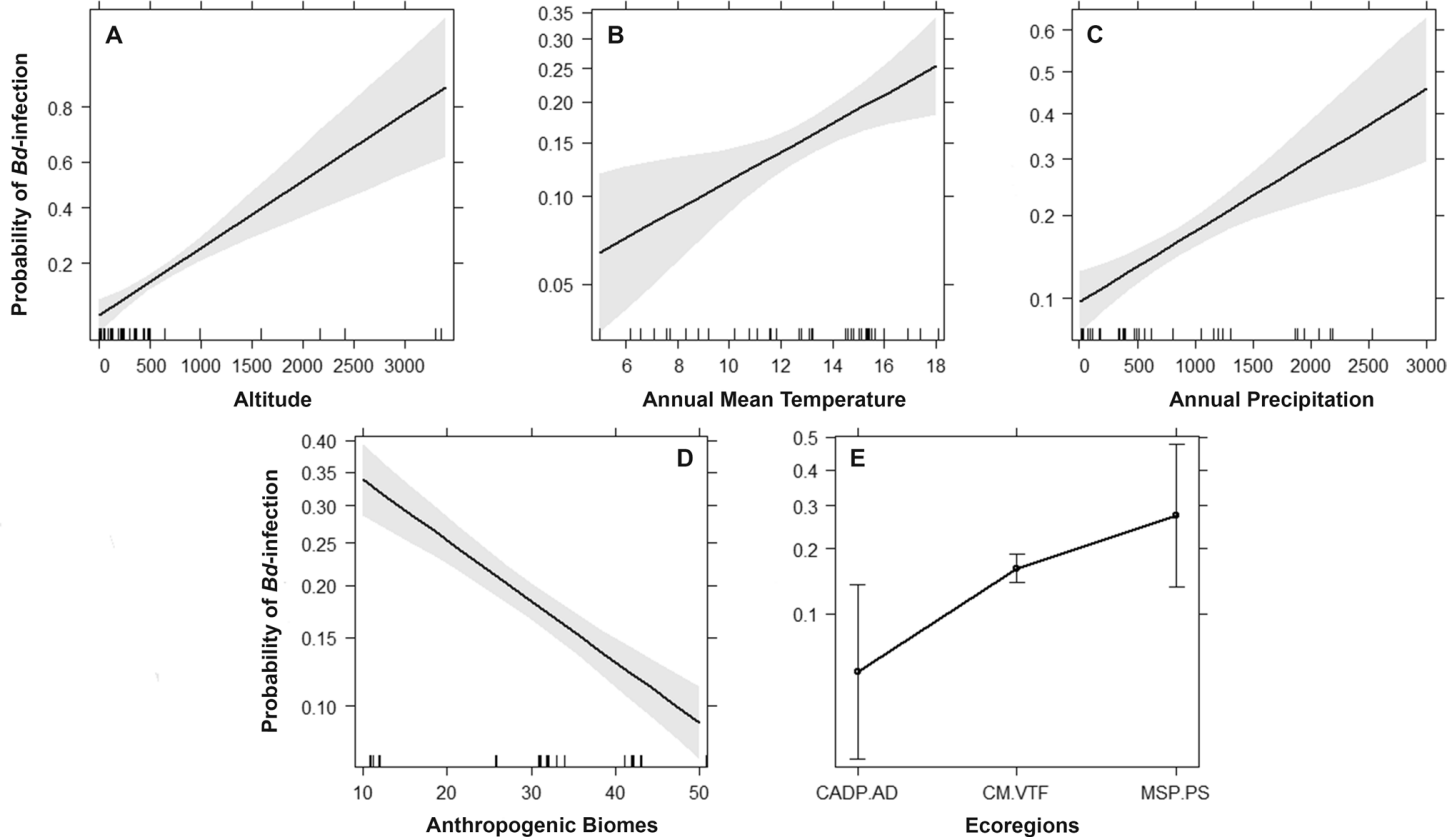
Best fit generalized linear model for Batrachochytrium dendrobatidis (Bd) prevalence in Chile: Bd prevalence ~ altitude + annual mean temperature + annual precipitation + anthropogenic biomes + ecoregion. Separate effects of (**A**) altitude, (**B**) annual mean temperature, (**C**) annual precipitation, (**D**) anthropogenic biomes and (**E**) ecoregions are shown. Line was draw using estimates from best supported generalized linear model. Grey shading is 95% confidence intervals. Hash marks in the x-axis represents measured values. Abbreviations in (**E**), CADP.AD = Chilean Andean dry puna + Atacama Desert, CM.VTF = Chilean matorral + Valdivian temperate forest, MSP.PS = Magellanic subpolar forest + Patagonian steppe.

From the models tested to evaluate *Bd* infection risk against bioclimatic and anthropogenic factors (Table S2), the best model included the variables: altitude, annual mean temperature, annual precipitation, anthropogenic biomes and ecoregions (AICw = 0.88, AICc = 424.3, Z = − 4.9; d.f. = 42, Table S3) *Batrachochytrium dendrobatidis* infection probability was positively correlated with altitude (95% CI 1–1.01), annual mean temperature (95% CI 1.03–1.22), and annual precipitation (95% CI 1–1.01; Fig. 3A–C). Also, *Bd* infection was positively associated with anthropogenic biomes (95% CI 0.9–1; Fig. 3D), which means a higher *Bd* prevalence was observed in highly anthropogenic impacted ecosystems. Using model averaging, the anthropogenic biomes variable better explained *Bd* prevalence, and it was present in almost all the candidate models with low AICc scores (delta AICc < 4; Fig. 3D; Table S2). Also, the effect of ecoregions was significant, explaining differences in *Bd* prevalence (OR = 3, 95% CI 1.1–11.4, Fig. 3E, Table S3). Other factors, such as amphibian species richness and human footprint were not predictive for *Bd* prevalence.

### Spatial distribution of *Bd* clusters

Our spatial analysis detected eight statistically significant clusters of *Bd*-infection, all located in central-south Chile within the Chilean Matorral and Valdivian Temperate Forest ecoregions (Fig. 1C, Table 2). Half of the clusters, all of similar size, were located in the contiguous Metropolitan and Valparaíso administrative regions, the most densely populated area in the country. A large single cluster was identified further south, covering an area highly impacted by land-use change to exotic pine and eucalypt monocultures. The remaining three clusters were smaller in size and located near the cities of Angol, Valdivia and Puerto Montt, all in southern Chile. In the four spatial clusters in central Chile, the observed *Bd* prevalence was almost three times higher than would be expected by chance (Loglikelihood ratio range = 16.31–117.73 and relative risk range = 2–3.7). The Global Moran’s I index was statistically non-significant (p = 0.2).

**Table 2:** Spatial clusters of *Batrachochytrium dendrobatidis* infection in anurans from Chile studied between 2008 and 2018 ordered by significance.

Cluster	Latitude	Longitude	Radius (km)	O	E	O/E	RR	LLR
1	− 33.04	− 71.5	33.5	153	47.4	3.22	3.75	117.73
2	− 41.43	− 72.89	0	45	14	3.22	3.36	32.54
3	− 34.23	− 70.48	34.7	34	8.99	3.78	3.91	31.77
4	− 33.34	− 70.36	31.3	64	32.7	1.96	2.04	16.31
5	− 35.83	− 72.51	81.9	26	8.8	2.95	3.02	15.99
6	− 33.59	− 71.23	33.4	74	41.1	1.8	1.88	14.81
7	− 39.81	− 73.26	0	10	2.3	4.36	4.4	11.60
8	− 37.7	− 72.59	18.5	33	15.3	2.16	2.21	10.58

For each cluster area, observed cases (O), expected cases (E), observed/expected (O/E), relative risk (RR) and log-likelihood ratio (LLR) are shown.

## Discussion

Amphibian chytridiomycosis is well recognized as a main causative factor of the current global amphibian extinction crisis^10,57^. Therefore, it is essential to identify risk factors facilitating the occurrence of *Bd*, as well as high risk areas of infection, which can provide guidance for effective conservation management^58^. Our results show that, at the landscape level across a large latitudinal gradient in Chile, *Bd* occurrence is: (i) biased towards certain families and species that use aquatic habitats; (ii) largely determined by bioclimate and human associated risk factors such as altitude, annual mean temperature, annual precipitation and anthropogenic biomes; and (iii) grouped in spatial clusters associated with urban centres.

Overall, our data shows that *Bd* is widely distributed in Chile, infecting an extensive number of amphibian species and over broad altitudinal (0 to 3460 m) and latitudinal (18° to 48°S, comprising 3400 km) gradients. This included the finding of *Bd* infection in a Puerto Eden frog (*Chaltenobatrachus grandisonae*) near Villa O’Higgins (Fig. 1A), extending the previously known southernmost global record of *Bd* by 588 km further south^59,60^. From the total of 40 sampled anuran species in Chile (64% of total richness)^49^, we found that 24 species showed evidence of *Bd* infection (see Table 1). All 11 species which had a *Bd* infection prevalence > 30% were exclusively aquatic amphibians, likely due to a higher contact rate with the infective stage of *Bd* in the aquatic environment^53,61^. Of these aquatic species, high *Bd* prevalences were found at the extreme north of the Andes in the altiplano frogs *T. pefauri* (3308 m) and *T. chusmisensis* (3365 m; see Table 1)*.* Chytridiomycosis-related mortality has been described in the related species, *T. pisanoi* and *T. atacamensis* from northern Argentina^62^ and *T. marmoratus* from Peru^17,63^. In addition, *Bd*-implicated disappearances of *Telmatobius* populations have been described in Peru^17^ and Bolivia^64^.

The effects of *Bd* infection on anurans are highly variable and species-, population- or context-specific. For example, some species exhibit high disease-induced mortality, while others experience no detrimental individual or population effects of disease while maintaining enzootic infections^57,65^. In some cases, populations with cryptic, enzootic infections can experience chytridiomycosis-related mortality under certain environmental conditions, such as drought^41^. Vredenburg et al.^65^ proposed a 10,000 ZE infection load rule, above which lethal disease invariably occurs. In our study, the species with highest *Bd* loads were *C. gayi* (median 436 ZE and a maximum of 409,440 ZE) and *R. darwinii* (median 1170 ZE and a maximum of 84,709 ZE; Table 1), possibly indicating impacts at the population level in these species. We recommend, therefore, that longitudinal population monitoring and *Bd* surveillance programmes be initiated or continued for these threatened species at *Bd*-positive sites (e.g., see Binational Conservation Strategy for Darwin's Frog^66^). We found statistical differences in ZE between aquatic and terrestrial species, which emphasizes the role of aquatic species and aquatic environments in the maintenance and spread of *Bd*. Population declines due to *Bd* infection can occur in the absence of evident mass mortality or other obvious signs of disease, as evidenced by the *Bd*-driven declines of *R. darwinii* in Chile^53^ and the possible extinction of its sister species, *R. rufum*, which has not been observed since 1981^48,52,66^.

Our best ranked model included the effects of altitude, annual mean temperature, annual precipitation, anthropogenic biomes and ecoregions, as predictors of *Bd* infection (Fig. 3). Anthropogenically-disturbed ecosystems proved to be one of the most important predictors that explain *Bd* infection. Also, altitude, annual mean temperature and annual precipitation were positively associated with *Bd* prevalence. Amphibian chytridiomycosis has been reported at high elevations, for example in the Rocky Mountains^67^, the Sierra Nevada^65^, the Pyrenees^68^ and the high Andes^63^, suggesting that cold high-altitude environments do not necessary limit *Bd* spread and subsequent impacts on wild populations. The arrival of *Bd* in high altitude areas has been facilitated by human movement that has spread the fungus among isolated water bodies, but also climate change can facilitate such spread modifying the environment that anurans inhabit^63^ and further force the severity of infection^35^. In addition to altitude, temperature and precipitation appear to be relevant climatic variables shaping the occurrence of *Bd* in Chile, as previously reported for other world regions^21,27,36,43,69–73^. However, other studies have found sometimes different patterns (e.g.,^39,41,74^), suggesting that the mechanisms between these climatic factors and *Bd* occurrence are complex.

In our study, the highest prevalences of *Bd* infection were detected near to densely populated human settlements. Our results show that high human perturbation (anthropogenic biomes) is correlated with an increase in *Bd* infection probability, highlighting the importance of human activities on the epidemiology of *Bd*, possibly due to human-assisted pathogen introduction and spread (e.g., through the transport of *Bd* contaminated water and sediment) between anuran populations^24,57,75^. Additionally, human activities can spread *Bd* through the movement of infected amphibians, as has been shown to occur with amphibian trade, including the introduction of exotic amphibians^24,46,47,76–78^. Also, it has been proposed that the reduced connectivity among amphibian populations resulting from human perturbation of the environment might impact host skin microbiome, affecting the innate immunity in amphibian skin against pathogens^79^. Habitat fragmentation can also affect ecological (e.g., colonization/extinction, host physiology, etc.) and evolutionary (e.g., local adaptation, evolution of resistance/tolerance mechanisms, etc.) processes that can affect host–parasite interactions^19,79^. The development of increased susceptibility to infection through amphibian immunosuppression as a result of environmental contamination (e.g., pesticides) and habitat perturbation^80^ are also possible impacts of human activities that increase the persistence and spread of *Bd*.

Our models showed that anthropogenic impacts and climate variables could synergistically interact and exacerbate infection risk (Table S2). The mechanisms enabling such synergy remain unclear, but our results support anthropogenic disturbance as a driver of *Bd* infection risk. In this context, anthropogenic biomes are generalizations for the restructured terrestrial biosphere due to agriculture, forestry and urbanization^81^. Elevated risk of *Bd* infection in areas close to human activities and settlements has been described previously in both temperate and tropical regions^23,25,36,43,75,82^. Most studies based species distribution models of *Bd* in the Americas have found an association of *Bd* occurrence with several climatic variables, notably precipitation, temperature and seasonality^13,21,32,33,44^, although few incorporate explicitly the effect of human impact, such as urban centres, in the analyses^43^. Interestingly, Zumbado-Ulate et al.^33^ found a higher *Bd* occurrence in undisturbed ecosystems or protected areas, highlighting the fact that *Bd* occurrence is context specific as can be influenced by many factors including time of *Bd* introduction, species and population susceptibility, among others.

As with prevalence, the same association has been shown with intensity of infection, namely a positive association of *Bd* loads with anthropogenic disturbance^71^. The highest observed *Bd* prevalence was in the Chilean Matorral ecoregion, an area considered as a priority for global biodiversity conservation^83^. This region harbours a high level of anuran endemism^49^, yet contains the highest human population density in the country (almost 90% of the Chilean population). Consequently, increasing urbanization is resulting in deforestation and habitat loss which is negatively impacting amphibian populations^84^. Such environmental changes could favour the invasion of alien species, including *Bd*^24^.

All clusters of *Bd*-infection were located in central-south Chile, suggesting that amphibians in this region are at a higher risk of *Bd*-infection than elsewhere in the country. Although these findings are similar to those found by Bacigalupe et al.^43^, by using a different approach in a much wider area of Chile, this strengthens the hypothesis of urban centres playing an important role in the epidemiology of *Bd*. The four clusters located in the Metropolitan and Valparaíso regions could result from a potential initial introduction of *Bd* in this part of Chile and its subsequent spread^46^ with fewer clusters in the south of Chile. When *Bd* is introduced to a new geographic location, first foci of introductions represented by narrow spatiotemporal cluster(s) occur followed by subsequent spread over time^68,85^. This has been seen in Spain, where *Bd* infection shows a pattern of introduction and spread along the Pyrenees with narrow spatial clusters, indicating recent introductions into Iberian biomes^35^. Our results are consistent with such a pattern having occurred in our study area. An alternative hypothesis is that instead of high-risk areas we might be capturing oversampled regions^86^. Therefore, we recommend considering other methods such as species distribution models^43^ or kriging interpolation^33^ to have a more accurate picture of the identified high-risk areas.

*Batrachochytrium dendrobatidis* has been recognized as causing the greatest recorded loss of biodiversity due to a single pathogen^10^. Therefore, having a better understanding of the factors that shape the occurrence of *Bd* at the landscape level is valuable for conservation strategies and actions at the regional or country levels to halt the loss of biodiversity due to chytridiomycosis^14,87,88^. In this study, we provide evidence linking climate and anthropogenic factors as macroscale drivers of *Bd* occurrence at the landscape level. In particular the interaction of altitude, temperature, precipitation and human modified landscapes, appears to be the most relevant factors facilitating establishment and spread of *Bd* over large areas. Although we did not observe any mass mortalities or obvious signs of disease, we found high infection loads (> 10,000 ZE) which have been shown to lead to fatal chytridiomycosis in other amphibian species, with potential impacts at the population level. Although some species can coexist initially with high loads of *Bd*, host defence mechanisms such as anti-*Bd* skin microbiota, immunity, and skin peptides could reduce Bd infection loads to allow host–pathogen co-existence^89,90^. Strategies are required to prevent the further spread of *Bd* and to mitigate its impacts where it already occurs^10^. Our study provides valuable information for the design of such conservation strategies as long-term monitoring and control of biotic and abiotic factors (e.g., environmental disinfection, anti-*Bd* microbiome bioaugmentation or environmental management to reduce *Bd* exposure)^91,92^ in areas with high occurrence of *Bd* and especially in areas where amphibian populations are under a high degree of anthropogenic pressure.

## Materials and methods

### Ethic statement

This study was approved by Bioethics Committees of the Universidad Andres Bello (reference number 13/2015) and the Zoological Society of London’s Ethics Committee (WLE717), and followed the guidelines under permit from the Chilean Agriculture and Livestock Service (351/2015). All methods are reported in accordance with ARRIVE guidelines (https://arriveguidelines.org).

### Study area and sampled amphibians

From 2008 to 2018 we sampled amphibians at 162 sites from north (18°11′43″S, 69°34′6″W) to south (51°23′27″S, 72°46′59″W) Chile, covering a latitudinal gradient of 3700 km and an altitudinal range from sea level to 4434 m. We sampled recently metamorphosed, juvenile, and adult frogs of 40 species belonging to seven families from sites representatives of all the six ecoregions present in Chile: Andean Dry Puna, Atacama Desert, Chilean Matorral, Valdivian Temperate Forest, Patagonian Steppe, and Magellanic Subpolar Forest^93^. Since in Chile many amphibians are inactive during winter, for *Bd* prevalence study sites were surveyed only once in spring–summer and at each site a minimum of 23 amphibian samples were obtained. Minimum sample size was calculated assuming a test sensitivity of 100%, expected *Bd* prevalence of 12.5%^52^ and level of confidence of 95%.

### Animal capture, biosecurity and sampling

Each amphibian was located through diurnal and nocturnal captures by direct observation and caught by hand or, in the case of aquatic species (i.e., *Calyptocephalella gayi*, *Telmatobius* spp. and *X. laevis*), caught using herpetology nets or funnel traps baited with chicken liver^94,95^. Following capture, each individual was handled for sampling with the use of clean disposable nitrile gloves and then released back to the exact point of capture. To minimize any false positive results and to avoid pathogen cross-contamination within or between study sites, a strict field sampling and disinfection protocol was followed^20^. For *Bd* detection, a non-invasive skin swab (MW100, Medical & Wire Equipment Co.) was obtained from each amphibian by firmly running it five times each over the ventral abdomen, the pelvis, both ventral hind limbs (femur and tibia), and the plantar surface of both hind feet, to complete a total of 35 strokes^52^. Swabs were kept in a refrigerated box until being stored frozen at – 80 °C once back at the laboratory until they were analysed.

### *Bd* detection assay

Extraction of DNA from skin swabs and subsequent detection of *Bd* DNA using a specific real time qPCR assay was done^20^. For each sample, diagnostic assays were performed in duplicate, and standards of known zoospore concentration were included within each PCR plate as positive controls. We assumed that a *Bd*-positive swab indicated *Bd* infection. By including known concentrations of *Bd* DNA in serial diluted positive control (four standards of 100, 10, 1 and 0.1 *Bd* genomic equivalent) wells on each PCR plate, we were able to quantify infection intensity, which we defined as the number of zoospore equivalents/swab (ZE). To quantify and correct the infection intensity per swab, each genomic value was multiplied by 120 following Hudson et al.^96^.

### *Bd* prevalence and infection loads by family, host ecology and ecoregion

We first calculated prevalence by counting the number of positive animals in a particular taxonomic family, host ecology (aquatic vs. terrestrial), or ecoregion divided by the total number of samples within that category. Host ecology was defined by considering whether the adult frogs of each species spent most of their time in or out of the water (see Table 1). We estimated 95% binomial confidence interval (95% CI) with a logistic (logit) parameterization for each category using the binom.confint function (R package ‘binom’) in the statistical software R v.3.1.3^97^. We evaluated whether there was a trend in *Bd* prevalence over time using a binomial generalized linear model (GLM) using year as an explanatory variable. The deviance of a null GLM model was estimated to explore the contribution of time (year) as an explanatory variable. An autocorrelation function ‘acf’ was used to explore a potential temporal autocorrelation of the residuals. Finally, we applied odds ratio (OR) statistics to estimate the probability the amphibian to having *Bd* at each site in different years.

### Modelling *Bd* prevalence across the landscape

We employed an information-theoretic modelling approach to contrast the adequacy of different working hypotheses explaining the geographic occurrence of *Bd* infection in our landscape gradient. In order to model *Bd* infection, we used bioclimatic and anthropogenic factors as explanatory variables and *Bd* prevalence from each of the 162 surveyed sites as response variable. Eight variables derived from landscape-scale geographic layers were used as predictors in the statistical modelling. Explanatory variables included annual mean temperature, temperature seasonality, annual precipitation, altitude, human footprint, anthropogenic biomes, ecoregions, and amphibian species richness (see Table S1 for a full description of each variable and data sources^81,98–103^. We extracted all data for each sampled anuran to GPS coordinates using raster layers of 30 s (~ 1 km^2^) spatial resolution with QuantumGIS v.3.8.2^104^. The assumption of normality of the data, the presence of outliers, the number of absolute zeros in the response variable and the collinearity between environmental variables and anthropogenic factors were explored. We only retained those variables with a correlation coefficient < 0.7 by the Pearson and Kendall tests. The homogeneity of variance was verified using the residuals of the model, plotting residuals vs. fitted values, and making a similar set of conditional boxplots for the residuals^105^.

Twenty-one GLMs were constructed based on biological hypotheses using a binomial error structure (link = logit) for *Bd* infection model to evaluate factors that influence the occurrence of *Bd* infection at the individual level (i.e., *Bd* prevalence as response variable by study site). Then, we evaluated the degree of support for each competing model based on Akaike information criterion (AIC), which takes into consideration the likelihood of each model while penalizing for the number of included parameters to obtain the best-fitted model, and we used Akaike weights (AICw) to quantify the relative support of each model in the set (R package ‘MuMIn’). As a result, a suitable model that describes the factors associated with *Bd* prevalence in anurans was obtained. To validate the GLM final model, the residuals were plotted against fitted values to assess homogeneity. In addition, we plotted a histogram and q-q plot of the residuals to verify normality of the data (Figure S1). The existence of patterns in the residuals due to the assumption of independence was verified with a plot of the residuals against each of the explanatory variables. Finally, the effect of each significant variable in the GLM was displayed with R package ‘effects’^106^.

### *Bd* spatial cluster analysis

Each sampled site was categorized as *Bd*-positive or *Bd*-negative according to the results of the *Bd* qPCR assays: a site was considered positive if at least one individual swab sample tested positive. Visualization of the sample sites was carried out using QuantumGIS and projected for analysis using the WGS 1984 datum as a coordinate system. Spatial distribution was characterized by the Moran’s I spatial autocorrelation^107^, to identify spatial autocorrelation globally. We used Kulldorf´s clustering algorithm^108^ under Bernoulli probability model, using the software SatScan v.9.4.4^109^ to identify any cluster of *Bd*-positive samples across space with the proportion of infection at a given sample site. The model was run using *Bd* locations under the null hypothesis that cases were randomly distributed in space. The model was set to scan for areas with high *Bd*-positives numbers to test for clusters with a spatial occurrence higher than that outside the cluster. Briefly, the number of observed and expected *Bd*-infected amphibians is counted by a scanning window that moves across space for each location and variable window sizes^108^. Scan statistics allows the detection of the most “unusual” excess of observed *Bd*-positives and therefore provides georeferenced high-risk areas of *Bd* infection. Distributions of the likelihood ratio and its corresponding *P*-value were obtained using Monte Carlo simulation by generating 999 replications of the data set under the null hypothesis. The test statistics were computed for each replication and the test was deemed significant at *P* < 0.05.

## Supplementary Information

Supplementary Information.
