# Supplementary material for: Bioclimatic and anthropogenic variables shape the occurrence of Batrachochytrium dendrobatidis over a large latitudinal gradient
Source: Sci Rep. 2021 Aug 30;11:17383. doi: 10.1038/s41598-021-96535-w (PMC8405646; doi:10.1038/s41598-021-96535-w)

**Table S1.** Explanatory variables used in the generalized linear model (GLM) of Bd prevalence of anurans at all sites from Chile.

|  |  |  |  |  |
| --- | --- | --- | --- | --- |
|  | **Variable** | **Description** | **Source** |  |
|  | Temperature (°C) | Annual mean temperature representative of 1970–2000, with a resolution of 30-arc seconds. | Booth et al., 2014; Fick & Hijmans et al., 2017 |  |
|  | Temperature seasonality (standard deviation*100) | Temperature seasonality representative of 1970–2000, with a resolution of 30-arc seconds. | Booth et al., 2014; Fick & Hijmans et al., 2017 |  |
|  | Precipitation (mm) | Annual precipitation representative of 1970–2000, with a resolution of 30-arc seconds. | Booth et al., 2014; Fick & Hijmans et al., 2017 |  |
|  | Altitude | Digital elevation model with a resolution of 30 m. | ASTER, 2018 |  |
|  | Human Footprint | A composite index which characterizes human influence on the land based on accessibility, anthropogenic land use, population density, and infrastructure. | Sanderson et al., 2002 |  |
|  | Anthropogenic biomes | The terrestrial biosphere human-altered form (including land use and human population) defined by global patterns of sustained direct human interaction with ecosystems in a resolution of 1 km. Range from 10 (dense settlements) to 50 (forest with inconsequential human population). | CIESIN, 2005; Ellis & Ramankutty, 2008 |  |
|  | Ecoregion | Correspond to terrestrial ecoregions of Chile | Olson et al., 2001 |  |
|  | Amphibian richness | Obtained by overlaying the distribution range maps from the IUCN Red List Global Amphibian Assessment. | CIESIN, 2015 |  |
|  |  |  |  |  |

**Table S2.** Set of models accounting for the prevalence of *Batrachochytrium dendrobatidis* infection among anurans of Chile. Loglikelihood (logLik), Akaike information Criteria corrected for small samples size (AICc), AIC weight (AICw) and delta for binomial models with the prevalence of *Batrachochytrium dendrobatidis* (*Bd*) as response altitude (alt); annual mean temperature (temperature); annual precipitation (pp); anthropogenic biomes (biomas); ecoregion (Eco1); anuran richness (richglobal) and temperature seasonality (tempseason).

| **Predictors** | **df** | **AICc** | **delta** | **AICw** | **logLik** |
| --- | --- | --- | --- | --- | --- |
| alt+temperature+pp+biomas+Eco1 | 7 | 424.3 | 0 | 0.885 | -203.530 |
| alt+pp+biomas+Eco1 | 6 | 429.4 | 5.19 | 0.066 | -207.557 |
| alt+temperature+pp+biomas+Eco1+richglobal+tempseason | 9 | 430.2 | 5.93 | 0.046 | -203.368 |
| alt+pp+biomas | 4 | 437.2 | 12.92 | 0.001 | -214.066 |
| alt+biomas+Eco1 | 5 | 437.8 | 13.59 | 0.001 | -213.112 |
| alt+temperature+biomas+Eco1 | 6 | 439.7 | 15.42 | 0 | -212.672 |
| alt+biomas | 3 | 443 | 18.71 | 0 | -218.177 |
| pp+biomas+Eco1 | 5 | 453.1 | 28.82 | 0 | -220.728 |
| biomas+Eco1 | 4 | 454.2 | 29.9 | 0 | -222.556 |
| temperature+biomas+Eco1 | 5 | 456.5 | 32.22 | 0 | -222.428 |
| alt+temperature+pp+foot | 5 | 466.6 | 42.3 | 0 | -227.467 |
| alt+temperature+pp+foot+Eco1 | 7 | 469.7 | 45.47 | 0 | -226.264 |
| alt+temperature+pp+foot+Eco1+richglobal+tempseason | 9 | 471.9 | 47.63 | 0 | -224.217 |
| alt+pp+foot+Eco1 | 6 | 476.9 | 52.61 | 0 | -231.267 |
| alt+pp+foot | 4 | 479.4 | 55.13 | 0 | -235.167 |
| alt+foot+Eco1 | 5 | 483.6 | 59.34 | 0 | -235.987 |
| alt+temperature+pp+Eco1 | 6 | 487.3 | 63.02 | 0 | -236.471 |
| pp+foot+Eco1 | 5 | 488 | 63.78 | 0 | -238.209 |
| foot+Eco1 | 4 | 489.8 | 65.5 | 0 | -240.356 |
| alt+temperature+pp+foot | 5 | 492.3 | 68.02 | 0 | -240.328 |
| alt+pp+richglobal+tempseason | 5 | 509 | 84.74 | 0 | -248.691 |

**Table S3.** Coefficients of our best binomial generalized linear models with prevalence of *Bd* in Chilean amphibians as response variable. Estimates of Intercept, Standard Error, z value and p-value with the prevalence of *Batrachochytrium dendrobatidis* (*Bd*) as response altitude (alt); annual mean temperature (temperature); annual precipitation (pp); anthropogenic biomes (biomas) and ecoregion (Eco1).

| **Predictors** | **Estimate** | **Standard. Error** | **z value** | **p-value** |
| --- | --- | --- | --- | --- |
| **(Intercept)** | -4.3416421 | 0.8858618 | -4.901 | 9.53E-07 |
| **alt** | 0.0012334 | 0.0002223 | 5.547 | 2.90E-08 |
| **temperature** | 0.117361 | 0.0419038 | 2.801 | 0.0051 |
| **pp** | 0.0006849 | 0.0001621 | 4.224 | 2.40E-05 |
| **biomas** | -0.0418823 | 0.0053008 | -7.901 | 2.77E-15 |
| **Eco1CM.VTF** | 1.562581 | 0.5631441 | 2.775 | 0.00552 |
| **Eco1MSP.PS** | 2.119055 | 0.752406 | 2.816 | 0.00486 |

**Figure S1.** Autocorrelation to explore a potential temporal autocorrelation of the residuals of our best binomial generalized linear models with prevalence of *Bd* in Chilean amphibians as response variable.


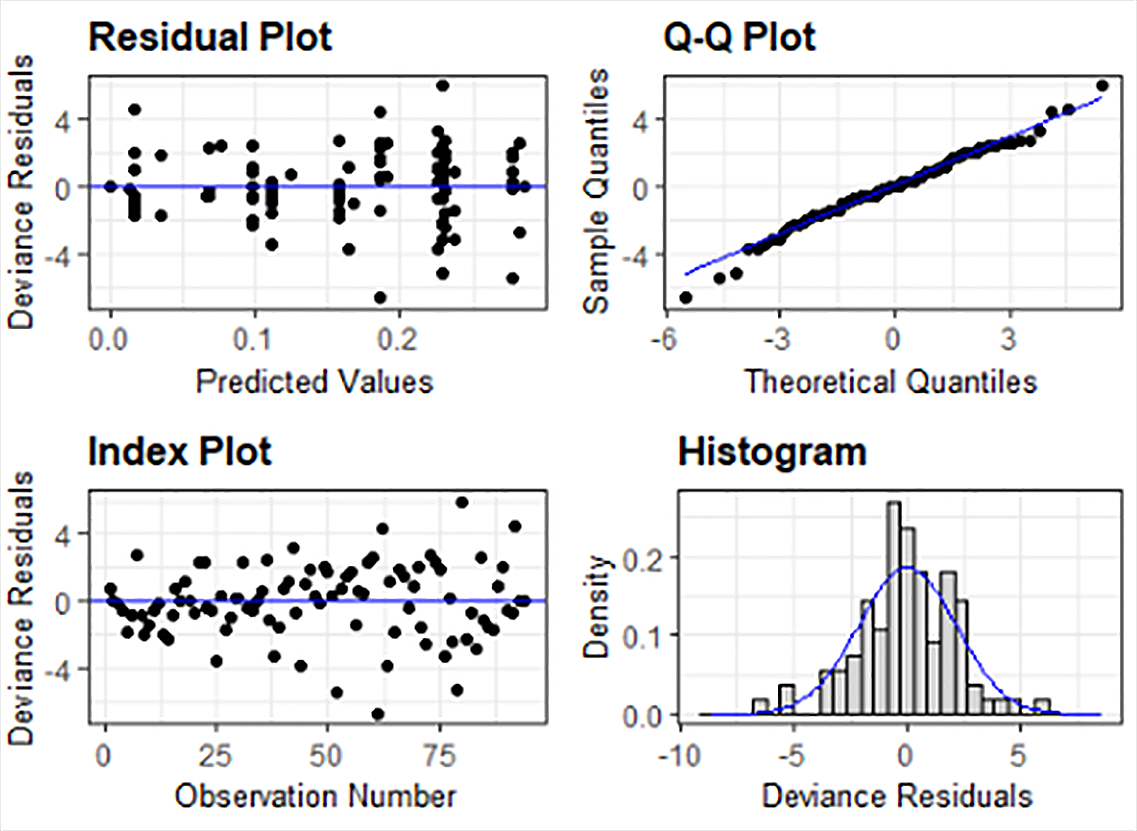

Supplement: Supplementary file 1 — Supplementary Information. [file 41598_2021_96535_MOESM1_ESM.docx]
